# Developing a Breast Cancer Screening Program in Nigeria: Evaluating Current Practices, Perceptions, and Possible Barriers

**DOI:** 10.1200/JGO.2016.007641

**Published:** 2017-01-25

**Authors:** Olalekan Olasehinde, Carla Boutin-Foster, Olusegun I. Alatise, Adewale O. Adisa, Oladejo O. Lawal, Akinbolaji A. Akinkuolie, Abdul-Rasheed K. Adesunkanmi, Olujide O. Arije, Thomas P. Kingham

**Affiliations:** **Olalekan Olasehinde**, **Olusegun I. Alatise**, **Adewale O. Adisa**, **Oladejo O. Lawal**, **Akinbolaji A. Akinkuolie**, **Abdul-Rasheed K. Adesunkanmi**, and **Olujide O. Arije**, Obafemi Awolowo University, Ile-Ife, Nigeria; **Carla Boutin-Foster**, State University of New York, Downstate Medical Center, Brooklyn; and **Thomas P. Kingham**, Memorial Sloan Kettering Cancer Center, New York, NY.

## Abstract

**Purpose:**

In low- and middle-income countries like Nigeria, women present with advanced breast cancer at an earlier age. Given the limited resources, development of screening programs that parallel resource capabilities of low- and middle-income countries is imperative. The objective of this study was to evaluate the perceptions, practices, and barriers regarding clinical breast examination (CBE) screening in a low-income community in Nigeria.

**Materials and Methods:**

A cross-sectional survey of women age 40 years or older in Ife, Nigeria, using multistaged sampling was performed. Information on sociodemographics, knowledge of breast cancer, screening practices, and willingness to participate in CBE screening was obtained using an interviewer-administered questionnaire.

**Results:**

A total of 1,169 women whose ages ranged from 40 to 86 years (mean age, 47.7 years; standard deviation, 8.79 years) were interviewed. The majority of women (94%) knew about breast cancer, whereas 27.5% knew someone who had had breast cancer, the majority of whom (64.5%) had died of the disease. Of the 36% of women who had breast screening recommended to them, only 19.7% had an actual CBE. Of these, only 6% had it in the last year. The majority of women (65.4%) were willing to have regular CBEs and did not care about the sex of the examiner in most instances. Lack of perceived need was the reason cited by women unwilling to participate.

**Conclusion:**

The majority of women were aware of breast cancer and knew it as a fatal disease. With the relatively encouraging number of those willing to be examined, a carefully designed CBE program coupled with advocacy to correct uneducated beliefs seems promising.

## INTRODUCTION

Breast cancer is a common cause of cancer-related deaths in most developing countries. With most patients presenting in advanced stages,^1^ it is not surprising that it is one of the most common causes of cancer mortality.^2^ Studies have shown a steady increase in the incidence of breast cancer in Nigeria from 15.3 per 100,000 in 1976 to 33.6 per 100,000 in 1992 to 52.1 per 100,000 in 2012.^3,4^

In developed countries, however, mortality from breast cancer has been on the decline despite the higher incidence of breast cancer.^5^ This is a result of early detection through organized screening programs and effective treatment modalities.^6-9^ In the United States, women with an average risk of breast cancer are recommended to undergo annual screening mammography starting at age 45 years and continuing up to age 54 years, after which they can transit to screening every 2 years or continue screening annually. It is also recommended that women between 40 and 44 years of age should have the opportunity to begin annual screening.^8^ However, the applicability of mammography-based screening programs is limited in low- and middle-income countries because of the challenges of poor infrastructure, poverty, and inadequate manpower. Waiting until such capabilities are developed, however, will lead to continued loss of life as a result of late presentation.

Therefore, clinical breast examination (CBE) has been recommended as a screening modality that may find usefulness in resource-poor settings while efforts are underway to attain the status of international best practices. Studies of programs in Africa and India^10-12^ provide a strong rationale for this assertion. The successes of these programs demonstrate the acceptability and feasibility for resource-limited countries to develop formal programs at various levels of health care delivery that use CBE for screening either solely (where mammography is unavailable) or to complement mammography (where it is in short supply). However, this should be done cautiously, given previous experiences where most participants failed to comply with the screening recommendations after commencement of the program.^13^ As a first step in developing such a program, it is imperative to understand the peculiarities of the target community to conduct a successful, socially acceptable, and sustainable program. This study, conducted in a southwestern Nigerian community, set out to determine the perceptions, practices, and possible barriers of CBE-based screening program in a low-resource setting.

## MATERIALS AND METHODS

### Study Population

This study was conducted in the Ife Central local government area of Osun State, southwestern Nigeria, between February and April 2016. Ife Central local government is one of the two local governments in Ile-Ife, a city in southwestern Nigeria with a population of 167,254 (2006 census). It has a teaching hospital where specialized care, including breast services, is offered.

The local government is made up of 11 wards, each having a variable number of streets. Considerable variations exist in the social characteristics of the various wards; hence, we sampled on all the wards for equal representation. Sampling was done using a multistage stratified sampling technique first into wards and then into streets within each ward. The number sampled from each ward was proportional to the population size of the ward. Women age 40 years and older were eligible for the survey.

### Instrument

We used a 35-item, study-specific, interviewer-administered questionnaire first designed in English and later translated to Yoruba, the local language in Ile-Ife. Translation was done by a Yoruba language education expert. Pretesting was done using both English and Yoruba versions in a cohort of 20 women in Ile-Ife, and this was reviewed before the final adoption of the questionnaire. Interviews were conducted by a team of undergraduate and graduate students who were trained before commencement of the study and could effectively communicate in both English and Yoruba languages depending on the preference of the participant. Each interview lasted an average of 10 minutes. The questionnaire gathered information on demographic characteristics, breast cancer knowledge and experience, practice of CBE, willingness to participate in a CBE program, and possible barriers to participating in such a program.

### Key Variables

The key variables in this study were receipt of CBE, willingness to participate in a regular CBE-based program, and reasons for refusal to participate. We used two measures of receipt of CBE; these were receipt of CBE ever and receipt of CBE during the past year.

### Statistical Analysis

Sociodemographic characteristics, awareness of breast cancer, practice of breast cancer screening, willingness to participate in CBE screening, and other key variables were analyzed using descriptive statistics. Simple and multivariate Poisson regression with robust variance estimation was used to derive prevalence ratios with 95% CIs for the assessment of factors associated with willingness to participate in CBE. Variables were selected for the multivariate Poisson regression by the backward stepwise elimination method, with *P* = .25 set as the level for removal from the model. Data analysis was done using STATA version 12 (STATA, College Station, TX).

### Ethics

This study was approved by the ethical committee of the Institute of Public Health at Obafemi Awolowo University (Ile-Ife, Nigeria). Approval was also obtained from the Ife Central local government authority before the survey was conducted.

## RESULTS

### Sociodemographics

A total of 1,169 women were surveyed across the entire local government. Their ages ranged from 40 to 86 years, with a mean age of 47.7 years (standard deviation, 8.79 years). Most of the respondents had some form of education, with only 15.6% having no formal education at all. Most were married and were traders, as listed in Table 1.

**Table 1:**
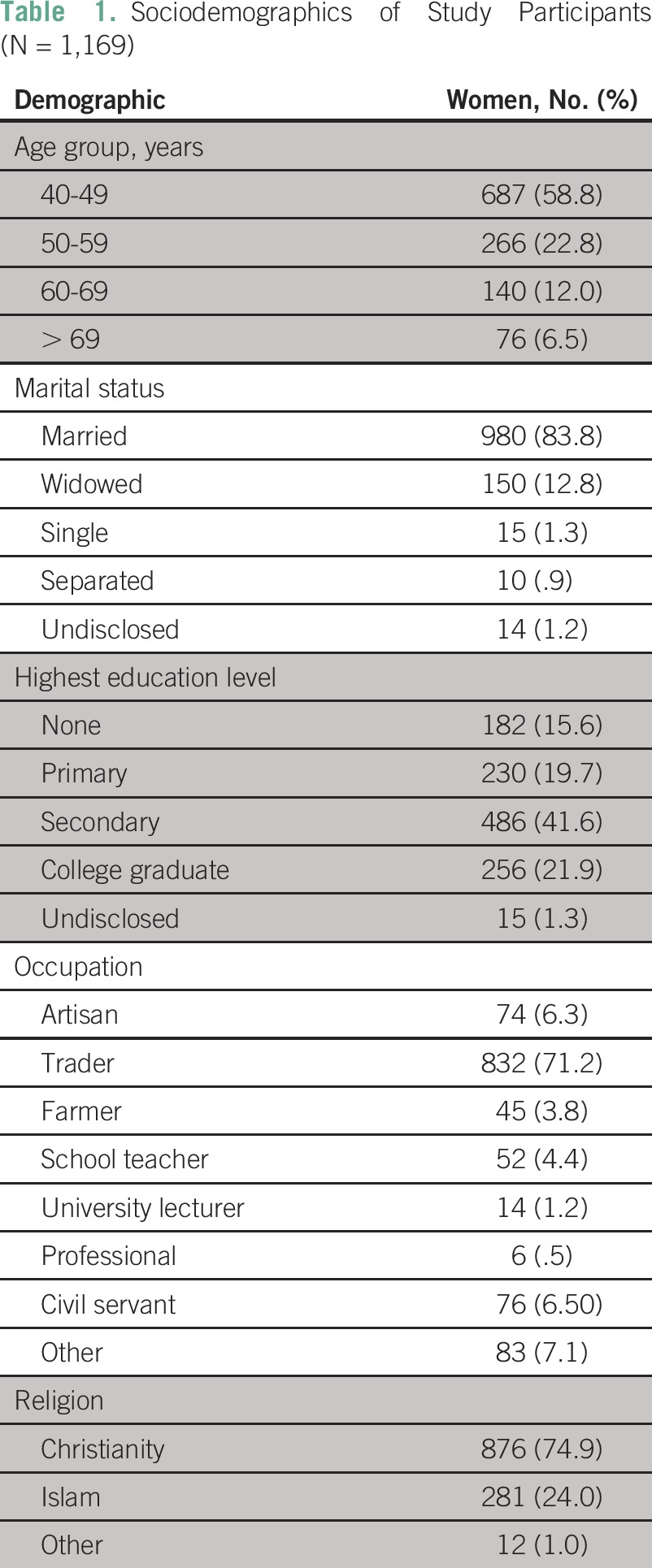
Sociodemographics of Study Participants (N = 1,169)

### Knowledge and Experience of Breast Cancer

Most of the respondents (94%) had heard about breast cancer, whereas 27.5% knew someone who had had the disease. Among these, 82.5% of respondents claimed the women they knew had received treatment in the hospital and that approximately two thirds of the women died of the disease (Table 2).

**Table 2:**
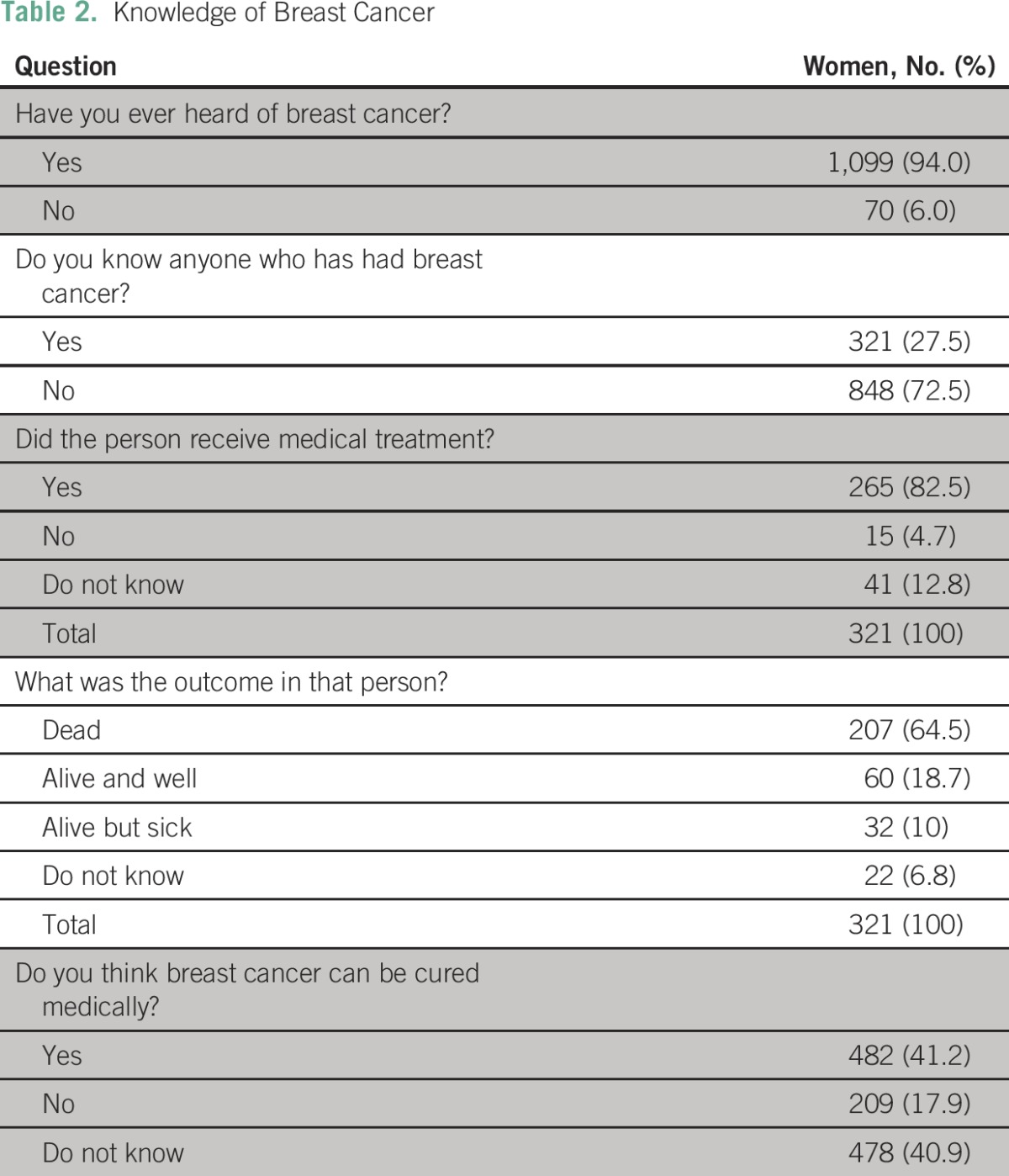
Knowledge of Breast Cancer

### General Health Behavior

Assessment of the respondents’ general health behavior showed that approximately two thirds of the respondents received treatment of their health issues in the hospital (63.9%), whereas the other respondents self-medicated (19.2%), visited pharmacies (9.2%), or patronized herbalists (3.7%). Other health behaviors assessed included receipt of a blood sugar test and Papanicolaou test. Only 42.8% of respondents had ever had a blood sugar test, whereas the remaining 57.2% had never had a blood sugar check. Papanicolaou testing for cervical cancer was performed in only 10.8% of the women, whereas the majority (89.2%) had never had a Papanicolaou test.

### Breast Cancer Screening Practice

Specifically, with regard to breast cancer screening, the majority of women had never been screened by any method. Only 37.7% of the respondents had ever had breast cancer screening recommended to them (Table 3) by a health worker (43%), mass media (37.8%), friends and relatives (13.6%), or religious and public seminars (5.6%). Regular breast self-examination was practiced by 31.2% of the women, whereas 23.5% claimed to examine their breasts irregularly and 45.3% did not examine their breasts.

**Table 3:**
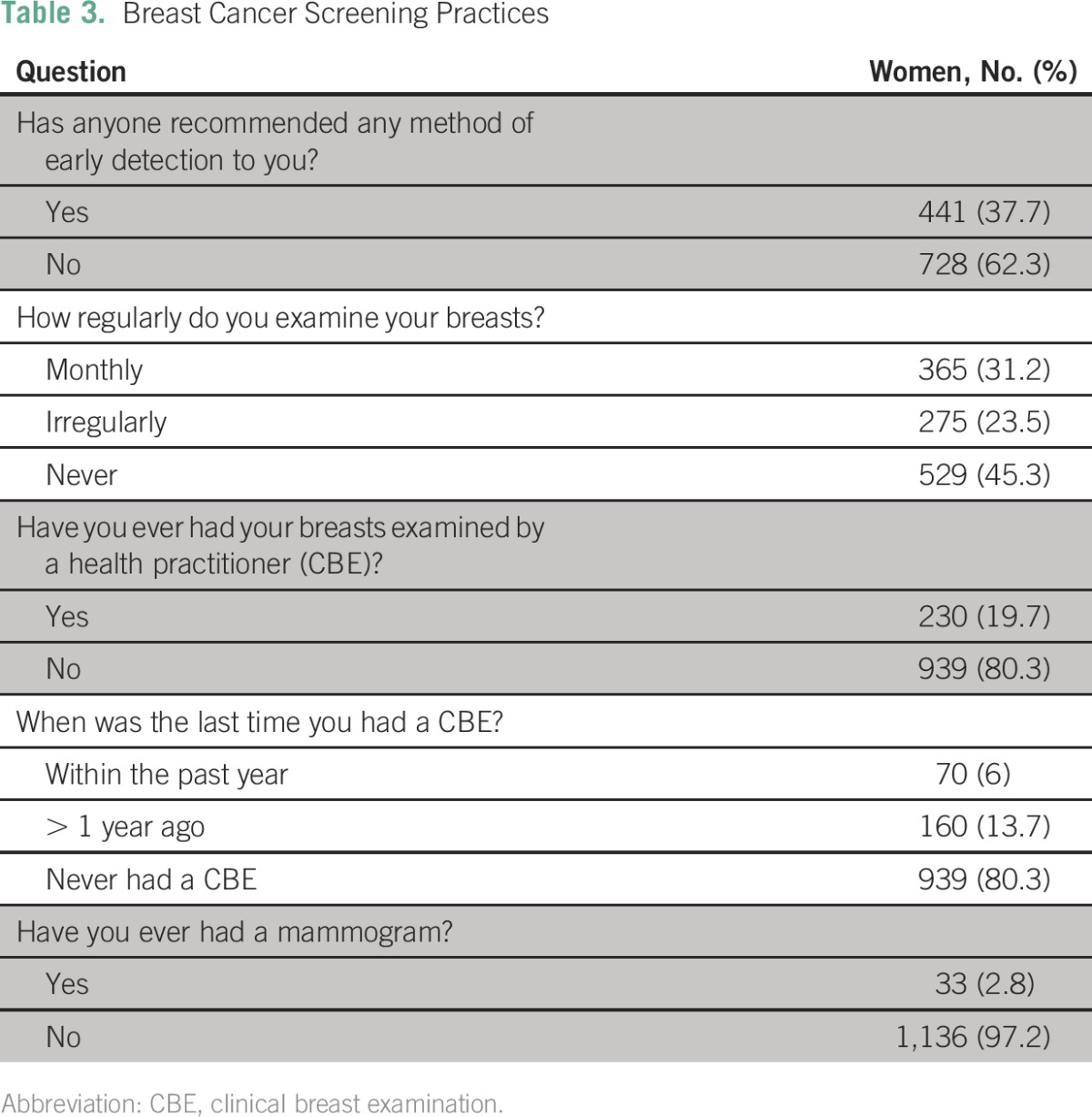
Breast Cancer Screening Practices

Only 230 women (19.7%) had ever had their breasts examined by a health practitioner. Of these women, only 6% had their breasts examined in the past year, 4.4% had their breasts examined in the past 2 years, and 9.2% had not had a breast examination in more than 2 years. Mammography screening had only been done in 33 women (2.8%).

### Attitude Toward CBE Screening Program

Seven hundred sixty-five respondents (65.4%) were willing to participate in a regular breast examination screening program, whereas 404 respondents (34.6%) were not. Lack of perceived need was the reason given for nonwillingness by those who declined. We examined preferences for the sex of the examiner and found that 249 women (32%) would prefer a female examiner and 15 women (2%) would prefer a male examiner; however, the majority of women (65.5%) did not care about the sex of the examiner. This was the case regardless of religion and level of education (*P* = .63 and *P =* .3, respectively). Prevalence ratios derived from Poisson regression with a robust estimate of variance showed that willingness to participate in CBE was significantly higher among women who were civil servants (adjusted prevalence ratio [APR], 1.21; *P* < .001), who knew someone who had breast cancer (APR, 1.15; *P* < .001), who practiced regular breast self-examination (APR, 1.22; *P* < .001), and who had a Papanicolaou test (APR, 1.20; *P* < .001).

## DISCUSSION

The current reality regarding the pattern of breast cancer presentation in most low- and middle-income countries is disturbing. Perhaps more worrisome is the lack of an organized effort to control this trend. Although the economic implications of a standard screening program are daunting, it is no excuse for the minimal efforts to date.

The use of CBE has been considered a surrogate for the standard screening modality in economically deprived countries and has been shown to produce good results.^5^ As a means of translating this into practice, we embarked on this study to obtain necessary information that may assist in developing a viable, culturally acceptable screening program. Our findings show that CBE uptake, although poor, is acceptable to the majority of the women without many social concerns.

Most of the respondents, who are women in their 40s, represent the population most affected by breast cancer in Nigeria and, indeed, most black populations.^1^ Although mammography is generally recommended in most instances for women older than age 40 years, survival benefit for women younger than age 50 years is controversial.^14,15^ Therefore, it may not be out of place to adopt CBE until capabilities are developed for routine mammography.

The fact that 94% of the respondents had heard about breast cancer is indeed a testimony to the relatively frequent occurrence of breast cancer in our society. However, one wonders what kind of knowledge is entrenched in the community given the high number of respondents who knew someone who died of breast cancer despite having received treatment in the hospital. The fact that less than half of the respondents believed breast cancer can be cured medically further solidifies the notion that breast cancer is mostly perceived as an invariably fatal disease. Although most deaths are a result of late presentation, they are usually attributed to failed treatment, thus perpetuating the idea that death is the inevitable consequence of breast cancer regardless of time of presentation or treatment. This is an aspect of community education that must be strongly addressed during awareness campaigns. However, improvements in the outcome of breast cancer treatment will be the most convincing evidence to correct this misconception.

With barely a third of women having ever had any form of breast cancer screening recommended to them, it is not surprising that less than one fifth have ever had a CBE, with the majority of these women not having received CBE in the past year. Health workers certainly play a major role in creating breast cancer awareness and making screening recommendations to their patients. As shown in this study, the majority of the recommendations were made by health workers. Contacts made with health personnel during visits to the hospital for various health challenges can be used as a means of creating awareness and for opportunistic CBE. A link can also be created between breast health programs and other primary health programs such as maternal and child health services, thereby using such platforms for screening. This leverages already existing infrastructure without creating a separate program that requires mobilization of resources specifically for breast cancer. This is a cost-effective design in resource-constrained settings where the incidence of breast cancer is not high enough for a cancer detection rate that justifies the investment of huge resources on a vertical program. Such an approach has also been favored by other breast cancer experts.^16^ Combining breast and cervical cancer screening is another approach that can be used as a cost-effective and more comprehensive screening program for women. This has also been successfully practiced with some good results.^11^

Training personnel to effectively carry out breast examinations is key to the success of such a program, and this has been successfully demonstrated by interventional programs that have used such a model.^11,17^ Nurses and midwives involved in maternal and child health, who make regular contacts with women, have been suggested as ideal personnel to carry out such examinations.^16^

Although known to be relatively inexpensive without any requirement for technology, a CBE screening program will require funding for training and other running costs. However, the relatively lower cost of CBE compared with other screening modalities may serve as a basis for lobbying policymakers to incorporate it into the health insurance scheme in countries where such programs exists.

The success of any screening program ultimately depends on the number of women who are eventually treated among those who screen positive. This can be enhanced by developing an effective referral system for diagnosis and treatment. An encouraging treatment completion rate of 92% was observed in a randomized controlled trial of breast and cervical cancer screening in India where such a referral system was in place.^11^ The concept of patient navigation, which was first described in 1990 by Harry Freeman in the United States, has also continued to find more relevance in promoting prompt diagnosis and treatment of screen-positive women. It is a patient-centered health care service delivery model that assists individuals, particularly the medically underserved, in overcoming personal, logistical, and system barriers to care across the cancer care continuum.^18,19^ A similar concept may also be of benefit in low- and middle-income countries where access to health care constitutes a challenge.

Because the breast is a private area of the body and because of the various social and religious factors that may hinder the acceptance of CBE, seeking the opinion of the target population about such intervention becomes imperative. This is particularly important because approximately 99% of women respondents in our study ascribed to some religious affiliation. It is encouraging that more than two thirds of the respondents were willing to have regular CBEs. Responses from women who declined such examination suggest that with proper education and awareness, many more women are likely to be won over, given that the reasons for nonwillingness were related to lack of perceived need. Such perceptions are probably based on the misconception of the essence of screening as a test meant only for those who know they have the disease. Understanding the essence of screening should thus feature prominently during public education campaigns. Regarding women’s preferences for the sex of the examiner, it is quite interesting to note that the majority of women did not care about the sex of the examiner. This was the case regardless of age, level of education, or religion. This finding was also observed in a study from southern Nigeria that evaluated, among other factors, the impact of the examiner’s sex on breast screening practices.^20^ However, this finding may not be generalizable, bearing in mind the concept of hidden sociocultural and religious barriers. As such, it is recommended that early detection programs be implemented alongside educational programs coupled with modifications culturally appropriate to the target community.^21^

As previously mentioned, there is a great opportunity for trained health personnel to provide breast cancer advocacy and screening to women who visit hospitals for various health challenges. A hospital-based CBE screening program can be designed to target such women. Even if the detection rate is low, minimal resources would be expended and awareness would increase. Women who do not visit hospitals regularly may be reached through community outreach programs. The willingness of women to have a breast examination, the few social concerns, and the negligible cost of CBE make a carefully designed program seem promising. In addition to creating an opportunity for early detection, CBE also serves as a means of improving the general health-seeking behavior of the populace, which certainly creates the necessary ground work for optimal use of standard screening facilities when they become widely available.

A limitation of this study is the lack of information on the perceptions of women and possible decisions that may be made in the event of a positive finding. Incorporating such information into prescreening awareness campaigns may promote compliance among those who screen positive. Another limitation is the homogeneity of the sampled population in terms of culture. Perhaps a more culturally diverse population reflecting the multiethnicity of Nigeria would make findings from this study more generalizable.

In conclusion, our findings show that CBE practice, although poor, is acceptable to the majority of women in the studied population with few social concerns. Creating awareness with educational programs is needed to correct erroneous perceptions about breast cancer and the need for screening.
